# Is there a place for ultrasound in diagnosing sarcopenia?

**DOI:** 10.2478/raon-2025-0035

**Published:** 2025-06-16

**Authors:** Tadej Rondaij, Nada Rotovnik Kozjek, Cene Jerele, Taja Jordan

**Affiliations:** 1 Institute of Physiology, Medical Faculty, University of Ljubljana, Ljubljana, Slovenia; 2 Department of Internal Medicine, Medical Faculty, University of Ljubljana, Ljubljana, Slovenia; 3 Department of Clinical Nutrition, Institute of Oncology Ljubljana, Ljubljana, Slovenia; 4 Institute of Radiology, University Medical Center Ljubljana, Ljubljan, Slovenia

**Keywords:** sarcopenia, ultrasonography, muscle mass, muscle quality

## Abstract

**Background:**

Sarcopenia is a progressive and generalised skeletal muscle disorder which presents as loss of muscle mass and function and is associated with increased likelihood of adverse outcomes, reduced quality of life and increased mortality. In developed countries, the prevalence of sarcopenia is rising due to increasing life expectancy. Still, in many clinical settings, sarcopenia may be overlooked and undertreated. While several tools are available for assessment of muscle mass and quality, there remains a need for safe, reliable and accurate diagnostic methods which can be implemented for both sarcopenia diagnosis and the evaluation of treatment efficacy.

**Conclusions:**

Ultrasound is an accessible and non-ionizing imaging technique that can potentially be used for that purpose. Several ultrasound parameters have been identified for their utility to provide assessment of muscle mass, quality and/or muscle function. Ultrasound is gaining recognition as an accurate and reproducible method of muscle mass assessment. However, there are still several limitations that preclude the application of ultrasound in routine clinical practice. Implementing a harmonized measurement protocol and conducting large-scale longitudinal studies on both healthy individuals and various patient cohorts could enable the establishment of clearly defined reference values for individual ultrasound parameters and, in turn, potentially reliable differentiation between normal and sarcopenic states.

## Introduction

Sarcopenia is defined as a loss of muscle mass and function, which affects 6–22% of older population^1^ and has a prevalence of up to 10% in the general population.^2^ Reduction in muscle mass may result from physiological (mostly hormonal) changes in advanced age, termed primary sarcopenia. Secondary sarcopenia may result from various pathological conditions or physical inactivity and is frequently associated with disturbances in the nutritional status, most notably malnutrition.^3^ Sarcopenia is now considered a muscle disease, with low muscle strength becoming the principal determinant, since muscle strength is superior to muscle mass in predicting adverse outcomes. Sarcopenia is associated with frailty, reduced quality of life, physical weakness, higher mortality, and increased healthcare costs.^4–8^

In clinical practice, sarcopenia is often overlooked and undertreated. Additionally, the reported prevalence of sarcopenia is highly dependent on the diagnostic method and on the criteria used for diagnosis.^9–12^ The European Working Group on Sarcopenia in Older People (EWSGOP2) propose that muscle strength should be used to assess whether sarcopenia is probable and then confirm the diagnosis based on reduced muscle mass and/or muscle quality. Since low physical performance predicts adverse outcomes, the degree of muscle function is proposed as an indicator of severity of sarcopenia.^8^ Even though extensive research regarding sarcopenia exists, accessible and accurate assessment of muscle mass and quality in a clinical setting remains challenging.

Muscle mass (quantity) can be estimated by various methods, with measurements usually adjusted for height or for body mass index (BMI). The most well-established radiological methods of measuring muscle mass are computed tomography (CT) and magnetic resonance imaging (MRI).^8,9,13–15^ Both methods provide accurate and reliable muscle mass measurements. However, several factors preclude the use of CT and MRI in everyday clinical practice for muscle mass and/or muscle quality measurement alone.^8,9,16^ Additionally, no consensus for cutoff points for sarcopenia has been reached regarding measurements of muscle quantity or quality obtained with either MRI or CT.

Dual-energy X-ray absorptiometry (DXA) is a more widely available instrument to non-invasively determine muscle quantity. Additionally, cutoff points for sarcopenia diagnosis have been established using DXA measurements. However, the method is relatively expensive, less accessible, and unportable.^9,17^ Additionally, results might not be consistent across different DXA instrument brands^15,18,19^, while the degree of concordance with gold standard techniques may depend on age and gender.^15,20^ DXA does not provide qualitative data regarding muscle tissue, which is increasingly valued in the assessment of sarcopenia.^21^ Since DXA examination is also associated with a small radiation dose, it is not suitable for all patients.

Bioelectrical impedance analysis (BIA) presents a relatively cost-effective and accessible method of body composition assessment, which is based on measuring the body’s resistance and reactance. The amount of muscle mass is calculated using prediction equations for a given population in lieu of being measured directly. The accuracy of results can therefore be affected by multiple factors, most notably the patient’s hydration status with fluid overload acting as a strong confounding factor.^22,23^

While more research has been made regarding the role of reduced muscle mass (muscle quantity) compared to changes in muscle quality in sarcopenic patients, it has become clear that muscle quality also plays a significant role in muscle function. Muscle strength has been shown to decline more rapidly than muscle mass, suggesting that age-related alterations of muscle composition may precede muscle mass reduction.^24–26^ Muscle quality may refer to muscle function (muscle strength or muscle power) per unit of muscle mass. However, it may be also interpreted as the relative presence of different components of muscle mass (e.g. muscle, vascular, fibrous and adipose tissue), thus referring to both micro- and macroscopic changes in muscle architecture and composition.^8,24,27,28^

Similarly to the definition of muscle quality, there is also no consensus regarding the most accurate assessment methods for muscle quality in routine clinical practice. CT and MRI are considered “gold standards” for non-invasive assessment of muscle quality. These examinations are used mostly in research settings and are not feasible to use in clinical practice exclusively for this purpose.^29–31^

There is an important and growing need for safe, non-invasive, accurate, cost-effective, and easily available methods that can provide information regarding both muscle quantity and quality, and that can be used in large population-based screenings.

Ultrasound (US) is an alternative method for assessing muscle mass and quality that is increasingly used in clinical practice for this purpose. US is a non-ionizing imaging technique that provides dynamic assessment of soft tissue structures, is portable, and highly accessible. A growing body of research shows that US is an accurate and reproducible method for measuring muscle mass in various populations.^9,15,32–35^ Ultrasound offers the advantage of evaluating individual muscles and muscle groups, a crucial capability given the growing evidence that age-related muscle mass decline varies significantly across different anatomical regions.^36–38^

The EWSGOP2 working group recognises ultrasound as an accurate method of muscle assessment, emphasize the advantage of this method as being able to assess both muscle quality and muscle quantity. However, the working group has also stressed the need for further research to confirm the validity of ultrasound in muscle assessment in patient populations with varying health conditions and functional status.^8^

Accordingly, ultrasound examination for this purpose still has several limitations. Currently, it is used primarily for research purposes and is not standardized. There are no established cutoff values for various parameters, and the number of studies conducted is limited, especially across different patient populations. Standardizing the methods is essential to enable extensive and comparative studies that can address these issues.

The aim of this review is to present an overview of the current knowledge in the field of ultrasound assessment of muscle mass, muscle quality and muscle function and to provide a comparison between ultrasound and reference methods of assessing muscle mass and muscle quality in clinical practice. Additionally, this review aims to highlight areas where further research is needed, as well as promote further awareness of ultrasound as a simple, accessible, and accurate method, which has the potential to be used in sarcopenia assessment and may contribute to earlier identification and treatment of this disease.

## Methods

To identify the most recent evidence regarding the use of ultrasound in assessing muscle quantity, quality and muscle function and regarding agreement between the ultrasound method and reference methods for muscle assessment, a comprehensive bibliographic search was performed in the PubMed/Medline database using the keywords “ultrasound”, “sarcopenia”, “muscle mass”, “muscle quantity”, “muscle quality”, “muscle function”, “comparison”, “computed tomography”, “dualenergy X-ray absorptiometry”, and “bioelectrical impedance”.

The following filters were used for search refinement: meta-analysis, systematic review, review article, multicentre study, randomised controlled trial and clinical trial. We limited the search to the publication period from 2010 to 2024 and to articles published in the English language. We focused on research from five areas: »Utility of ultrasound for assessing muscle mass, muscle quality and muscle function«, “Utility of ultrasound for diagnosing sarcopenia”, »Ultrasound parameters«, »Ultrasound measurement protocol« and »Agreement between ultrasound and reference methods”.

## Results

Using the above-described search method, 1332 articles were identified in the PubMed database. After application of search filters, 278 articles were selected for further review. Only articles with available abstracts were reviewed. The articles were identified as relevant if they addressed at least one of the five above-mentioned areas of research. An additional 220 articles were excluded based on relevance. The remaining 38 articles were included in this review.

### Ultrasound parameters used in muscle assessment

Various ultrasound parameters may be used to assess muscle mass and quality. The ultrasound parameters used in the research and clinical settings of sarcopenia management are muscle thickness (MT), muscle cross-sectional area (CSA), echo intensity (EI), muscle fiber pennation angle (PA) and muscle fiber length (FL). Additional parameters used in ultrasound muscle assessment include muscle volume (MV), muscle stiffness assessed through elastography, muscle contraction potential, and assessment of muscle microcirculation with contrast-enhanced ultrasound (CEUS).^39,40^ A summary of the most commonly utilised ultrasound parameters is shown in Table 1.

**TABLE 1.** Ultrasound parameters used in muscle assessment, with main advantages and limitations

Parameter	Definition	Site of assessment	Advantages	Limitations
Muscle thickness (MT)	Distance between the superficial and deep muscle fascia	Every muscular compartment (most studies on upper leg muscles)	Simple to measure High validity and reliability Demonstrated diagnostic accuracy for sarcopenia	Requires standardisation and fixed anatomic landmarks Unclear whether total body muscle mass can be estimated from MT
Anatomical crosssectional area (ACSA)	Area of the muscle perpendicular to its longitudinal axis at the point of the largest muscle diameter	Any muscle compartment which can be wholly visualised by ultrasound	Studies have shown high validity and reliability Demonstrated diagnostic accuracy of varying degrees for sarcopenia and low muscle mass	Requires standardisation and fixed anatomic landmarks ACSA and PCSA of larger muscle compartments might prove difficult to measure with conventional ultrasound methods and standard linear probes
Physiological crosssectional area (PCSA)	Area of the muscle perpendicular to the course of its muscle fibers at the point of the largest muscle diameter		Muscle strength can be inferred from PCSA	
Echo intensity (EI)	Median brightness of ultrasound image, expressed in gray scale (0–255)	Every muscle compartment	Provides information regarding the degree of intramuscular fatty infiltration Evidence of negative correlation with muscle function	Requires standardisation Measurements may be influenced by various external factors (e.g. ultrasound image settings, probe tilt, patient rest duration, participant positioning, patient’s hydration status, subcutaneous adipose tissue etc)
Fascicle length (FL)	Length of the fascicular path between the insertions of the fascicle into the superficial and deep muscle aponeuroses	Pennate muscles (mostly of the lower limb)	Provides information regarding the maximum force and speed of muscle fiber contraction Related to the force generating capacity of the muscle and muscle function	Requires specific operator training Accuracy of measurements are highly dependent on correct measurement technique (e.g. joint position, muscle contraction during measurement, probe placement on the skin, probe orientation relative to the muscle fiber course etc.
Pennation angle (PA)	Angle of insertion of muscle fiber fascicles into the deep aponeurosis			
Contrast enhanced ultrasound (CEUS)	Used to assess the degree of muscle vascularisation	Muscles of upper leg, most commonly quadriceps femoris	Provides information on changes of muscle vascularization, which has been shown to be a contributing factor in sarcopenia pathogenesis	Requires specific operator training and the use of contrast agents The utility of this method in the clinical setting is still unclear

Although an increasing number of studies incorporate various ultrasound parameters in muscle assessment, the most commonly measured parameters to assess muscle quantity and muscle quality remain muscle thickness and echo intensity, respectively. Due to their accessibility, size and location, upper and lower leg muscle groups, namely gastrocnemius and quadriceps femoris (particularly rectus femoris) are most widely measured. Additionally, most studies have shown that ultrasound parameters of these muscles are superior compared to other muscle groups regarding validity of the method to detect sarcopenia.^9,41^ The vast majority of studies have utilised linear transducer probes for muscle assessment, as is recommended by the European Geriatric Medicine Society (EuGMS) SARCUS (SARCopenia through UltraSound) working group, since linear transducer probes are more adapted to assess muscle anatomy. However, some studies have shown comparable reliability and validity of both curved and linear probes for select ultrasound parameters and muscle groups.^42,43^

### Ultrasound assessment of muscle quantity and agreement with reference methods

The most commonly used methods for assessment of muscle mass (quantity) both in clinical practice and research settings are magnetic resonance imaging (MRI), computed tomography (CT), dual-energy X-ray absorptiometry (DXA) and bioelectrical impedance analysis (BIA).

CT examination provides an accurate and reliable assessment of muscle mass and represents the gold standard for non-invasive muscle mass assessment. However, due to the associated high dose of radiation, it is not suitable for neither everyday clinical use in sarcopenia management, nor can the use of CT be justified for purely research purposes. DXA and BIA are more widely available, yet both methods present with certain limitations regarding either accuracy, cost-effectiveness and/or accessibility.

The following sections present an overview of ultrasound parameters, used in muscle quantity assessment, and of research on the agreement between these parameters and reference methods in sarcopenia diagnosis. Ultrasound parameters that have been used in muscle quantity assessment studies are muscle thickness, cross-sectional area and muscle volume.

### Muscle thickness and muscle volume

Muscle thickness is perhaps the most widely studied muscle ultrasound parameter in sarcopenia research. Muscle thickness represents the distance between the superficial and deep muscle fascia, while some authors define it as the distance between the bone-muscle interface and the adipose tissue-muscle interface.^44^ Muscle thickness is considered a reliable parameter for quantitative ultrasound muscle assessment^9^, which can be easily and quickly measured.

Several studies have confirmed good reliability and validity of ultrasound-measured muscle thickness compared to reference imaging methods (DXA, CT, MRI) and direct cadaver measurements.^9,45-50^ One study concluded that measurements of gastrocnemius medialis thickness obtained by ultrasound are reliable and correlate well with DXA-derived appendicular lean muscle mass and muscle performance in older individuals.^21^

In a study on patients with cirrhosis and sarcopenic obesity, Dhariwal *et al*. demonstrated that ultrasound-measured MT of thigh and forearm muscles demonstrate high diagnostic accuracy for sarcopenia and correlate well with computed tomography-determined skeletal muscle index (SMI) in these patients.^51^

In a recent comprehensive meta-analysis, Fu *et al*. concluded that ultrasound muscle parameters showed low to moderate diagnostic accuracy for sarcopenia, whereby the accuracy of the method depended on the parameters analysed, the muscles examined, the reference standards used, and the patient population included in the study. The authors of the analysed studies used different diagnostic criteria for sarcopenia, while almost all included studies used either DXA or BIA as the reference method for determining body composition. The MT of the gastrocnemius, rectus femoris and tibialis anterior muscles showed the highest, albeit moderate diagnostic accuracy for sarcopenia. The authors also reported cutoff values of ultrasound measurements of different muscle groups for sarcopenia diagnosis.^41^ Similarly, a meta-analysis conducted by Zhao *et al*. showed that the MT of the rectus femoris and gastrocnemius muscles showed the highest, yet also moderate, diagnostic value for low muscle mass or sarcopenia.^52^ A scoping review of six studies by Staempfli *et al*. also reported that the MT of rectus femoris showed the highest validity for sarcopenia diagnosis.^53^ Similarly, in a systematic review of six studies, Nies *et al*. concluded that US examination of the rectus femoris muscle is a promising method to aid in the diagnosis of sarcopenia in various clinical populations.^54^

A meta-analysis by Li *et al*., which included 9 studies using ultrasound, reported a high pooled correlation coefficient between MT of upper and lower limb muscles and DXA, demonstrating an acceptable diagnostic accuracy for sarcopenia.^55^

Despite promising results, further research is required to determine the general utility of US-measured muscle thickness in predicting sarcopenia. Some authors have suggested correcting the muscle thickness according to body mass or body mass index (BMI), since body weight may influence muscle thickness through increase of local adipose deposits.^56^ Regarding muscle volume, several prediction equations have been proposed based on measurable muscle ultrasound parameters and muscle volume determined by MRI.^57,58^ However, more studies are needed to correlate muscle thickness to total muscle volume of individual muscles using the proposed equations.

### Muscle cross-sectional area

The cross-sectional area (CSA) of the muscle depends on the number and size of individual muscle fibers and is usually determined at the point of the largest muscle diameter. It is important to distinguish between anatomical and physiological cross-sectional muscle area. The anatomical cross-sectional area (ACSA) is the area of the muscle perpendicular to its longitudinal axis, while the physiological cross-sectional area (PCSA) is the area of the muscle perpendicular to the course of its muscle fibers (ie, muscle volume divided by fascicle length). Anatomical cross-sectional area (ACSA) and physiological cross-sectional area (PCSA) are equivalent in non-pennate muscles.; however, in pennate muscles, they differ.^59^ Muscle strength is more closely related to PCSA than to ACSA because PCSA represents the maximum number of potential actin-myosin cross bridges that can be activated in parallel during contraction. Therefore, when assessing muscle strength, relying solely on ACSA measurements is not recommended.^59,60^ Muscle strength can be inferred from the cross-sectional area, as it correlates with muscle volume.^61^ Although an indirect assessment of muscle strength, it can prove useful in patients who are incapable of active muscle contraction.^62^

In a study involving a small group of healthy elderly individuals, Reeves *et al*. reported a strong correlation between the muscle CSA of the vastus lateralis muscle measured by ultrasound and that determined by MRI. The authors reported an intraclass correlation coefficients of 0.998 for the reliability of ultrasound and 0.999 for its validity when compared to MRI.^33^

Seymour *et al*. discovered a 25% reduction of rectus femoris CSA in patients with chronic obstructive pulmonary disease (COPD) compared to healthy controls. Additionally, they found a significant, though moderate, correlation between the CSA of the rectus femoris measured via ultrasound and the fat-free mass (FFM) derived from bioelectrical impedance analysis (BIA) in the control group (COPD patients).^63^

A meta-analysis conducted by Zhao *et al*. showed a moderate diagnostic accuracy of rectus femoris CSA for low muscle mass determined by DXA or BIA.^52^ Similarly, in a more recent meta-analysis, Fu *et al*. showed that the CSA of rectus femoris and biceps brachii muscles showed a moderate diagnostic accuracy for sarcopenia diagnosis.^41^ In a study on 313 geriatric outpatients, Ozturk *et al*. concluded that rectus femoris CSA may accurately predict sarcopenia in these patients (AUC 0.766 and 0.773 for women and men, respectively).^64^

A nationwide multicentre study on 991 hospitalised patients at risk for malnutrition demonstrated a significant positive correlation between the rectus femoris CSA and BIA-derived body cell mass as well as with handgrip strength, and a significant negative correlation with the Timed Up and Go test. Additionally, cutoff points of ultrasound measurements were determined for probable, confirmed, and severe sarcopenia.^65^ For several muscles/muscle groups, it might prove difficult to measure cross-sectional area with conventional ultrasound methods and standard linear probes. Extended field-of-view modes may be used to facilitate imaging in these cases.

### Ultrasound assessment of muscle quality and agreement with reference methods

As mentioned above, no consensus has been reached on the definition of the term muscle quality, which is used to describe both changes in muscle specific strength as well as the composition of muscle tissue. However, myosteatosis is the most commonly used indicator for muscle quality in both clinical practice and research setting. The term myosteatosis describes the pathological fatty infiltration of muscle tissue and is, independent from sarcopenia, negatively associated with survival and other adverse treatment outcomes in various patient populations.^66–69^ Muscle biopsy is considered the gold standard for assessing the degree of myosteatosis; however, due to its invasive nature and potential for complications, it is not used in routine clinical practice. CT examination is the most accurate non-invasive method for assessing myosteatosis, in which tissue attenuation is measured and expressed in Hounsfield units (HU).^70^

Several other novel indicators for muscle quality have been proposed, including BIA-derived phase angle. With the continued development and refinement of methods for assessing muscle tissue quality, these indicators are expected to become increasingly important in the diagnosis and management of sarcopenia.^8^

Ultrasound parameters used in the assessment of muscle quality are pennation angle, fascicle length and echo intensity. Additionally, muscle stiffness, determined by sonoelastography, contraction potential as well as assessing microcirculation using contrast-enhanced ultrasound may provide further information on muscle quality.

The following sections provide an overview of ultrasound parameters and techniques for muscle quality assessment as well as available data on agreement with reference methods.

### Echo intensity

Information regarding muscle composition may be obtained by measuring the echo intensity (EI) of the muscle, also termed muscle echogenicity.^71^ Increased EI is an indicator of muscle degeneration, which presents as an increase in the proportion of intramuscular fatty and connective tissue.^72^ Echo intensity is most often determined by analysing the intensity of image points of the US image using grey scale analysis. A histogram function is used in the analysis, which is enabled by several image processing programs. This type of quantitative grey scale analysis has been shown to be more accurate than visual subjective US image assessment.^73^ In a study on 40 young and older adults, Watanabe *et al*. demonstrated a moderate association between the echo intensity of anterior thigh muscles and CT-determined muscle attenuation of these muscles, signifying that echo intensity at least partially reflected the degree of intramuscular fatty infiltration.^74^

Based on several studies, echo intensity of lower limb muscles appears to be useful in sarcopenia detection. Isaka *et al*. found an association between echo intensity of the tibialis anterior and gastrocnemius medialis muscles and the presence of BIA-determined sarcopenia in older individuals.^75^ Similarly, Yamada *et al*. demonstrated the ability of echo intensity to differentiate between sarcopenia and normal/presarcopenia groups, diagnosed using BIA, in older men and women.^76^ In a study on 78 patients with rheumatoid arthritis, Yoshida *et al*. reported a superior discriminatory performance of combined EI and CSA measurements of biceps brachii and rectus femoris muscles in sarcopenia diagnosis, compared to EI and CSA measurements alone.^77^

Concerns have been raised regarding low interand intra-rater reliability of echogenicity measurements. Strasser *et al*. reported low intraclass correlation coefficients for EI, particularly in the older experimental group.^78^ However, several other studies have showed very good intraclass correlation coefficients (≥ 0.800) and very small standard errors of measurement (≤ 7.26 %).^57,79^ Valera-Calero *et al*. demonstrated good to excellent intra-(ICC 0.800–0.989) and inter-rater (ICC 0.841–0.948) reliability of EI measurements of cervical multifidus and short rotator muscles in healthy volunteers.^80^

The method of measuring EI has several limitations. The EI of muscles in the elderly population and in certain patient populations is significantly higher than in younger people, which must be considered in the final assessment.^81^ The assessment may also be influenced by various external factors, e.g. ultrasound probe parameters, probe tilt, patient rest duration, participant positioning, and the patient’s hydration status.^62^ Additionally, inconsistent methodological approaches used across studies measuring EI make comparing results challenging.

Fukumoto *et al*. studied the influence of focus depth of US images on the depth-dependent attenuation of EI and the relationship between EI and MRI-measured intramuscular adipose tissues (IntraMAT). The correlation between EI and IntraMAT was found to be stronger when the focus was kept in the middle of the rectus femoris or vastus intermedius muscle compared to the top of the image.^82^

Girts *et al*. determined that higher US image gain significantly increased EI values of the vastus lateralis muscle, whereas EI values were stable between depths of 4.0 and 6.0 cm.^83^ However, Paris *et al*. also showed the importance of maintaining consistent depth across all ultrasound measurements.^84^ Scafoglieri *et al*. demonstrated that even after standardising for gain, depth, and frequency, the EI values still differed significantly across other additional US settings.^85^

The effect of subcutaneous fat on EI measurement has also been the subject of numerous studies, with some conflicting results. Young *et al*. proposed a correction factor for in vivo subcutaneous fat thickness on EI measurements, which has been used in several subsequent studies.^86–88^ Müller *et al*. found that increasing exogenous fat thickness between the probe and the region of interest (ROI) resulted in a decrease in EI of the tibialis muscle.^89^ Contrarily, Palmer *et al*. found that raw EI correlated better than corrected EI measurements with physical performance in healthy older women.^90^

The exact influence of different US system settings and subcutaneous fat on EI measurements remains unclear. However, it seems imperative to use standardised settings as well as to assess the need to correct for subcutaneous fat thickness. Furthermore, more advanced image processing techniques, commonly referred to as texture analysis, have been suggested to potentially overcome some of the limitations linked to muscle echo intensity measurements.^91^

### Muscle fibre length and pennation angle

Muscle architecture can be described by the angle at which muscle fibers are connected to the fascia (pennation angle), as well as the length of the muscle fibers (fascicle length), both of which can be measured by US. The pennation angle was defined as the angle of insertion of muscle fascicles into the deep aponeurosis, while fascicle length was defined as the length of the fascicular path between the insertions of the fascicle into the superficial and deep aponeuroses.^59,92^

Muscle architecture plays an important role in muscle force generation and is related to muscle function.^93^ In sarcopenia, due to the smaller number of consecutive sarcomeres, the length of muscle fibers is reduced, while the pennation angle becomes smaller.^92^ Both parameters are related to a decrease in the maximum force and speed of muscle fiber contraction in sarcopenic patients.^94,95^

The measurements of muscle architecture parameters are highly dependent on correct measurement techniques. Joint position, muscle contraction during measurement, probe placement on the skin as well as probe orientation relative to the muscle fiber course may influence results.^96,97^ In cases where the fascicle extended beyond the boundaries of the acquired ultrasound image, the length of the missing portion can be estimated by linearly extrapolating both the observed fascicular trajectory and the aponeurosis.^59,92,98^ Concerning pennation angle measurement, significant variability may occur regardless of the measurement site, potentially due to differing levels of myosteatosis and/or fibrosis within the same muscle.^99^

Regarding calculation of fascicle length, several authors^57,100^ used the following formula:

FL( mm)=MT( mm)*sin(PA)−1,

where FL = fascicle length, mm = millimetre, MT = muscle thickness and PA = pennation angle.

One study reported the intra-rater correlation coefficient for the measurement of the pennation angle of the gastrocnemius medialis muscle ranging from 0.738 to 0.820.^21^ Several studies have shown good reproducibility of measurements in young individuals^101^, whereas Strasser *et al*. reported lower reproducibility of pennation angle measurements of the quadriceps muscle.^78^

The utility of muscle pennation angle measurement to differentiate sarcopenic vs. non-sarcopenic adults remains unclear. Similarly, while the fascicle length tends to shorten with advancing age, its capability of differentiating patients with and without sarcopenia is also questionable. In a study carried on 100 elderly community-dwellers, Kuyumcu *et al*. concluded that both sarcopenic and nonsarcopenic subjects had similar pennation angles of the gastrocnemius muscle, while muscle thickness and fascicle length values were significantly lower in patients with sarcopenia.^102^ In a study on 57 healthy elderly individuals, Alvarez *et al*. found no correlation between gastrocnemius pennation angle and DXA measurements, concluding that pennation angle is not a suitable measurement in sarcopenia diagnosis.^21^ However, in a study on 279 elderly and 60 younger controls, Narici *et al*. demonstrated a significant correlation between DXA-derived skeletal muscle index (SMI) and the ratio between FL and MT of the vastus intermedius muscle (termed ultrasound sarcopenia index, USI).^103^

As a measurement of muscle quality, the use of pennation angle in combination with other ultrasound parameters might provide a more comprehensive assessment of overall muscle health in sarcopenic individuals. The use of pennation angle in sarcopenia research remains relatively new, and further research is needed to validate its utility. Ultrasound measurement of fascicle length and pennation angle of pennate muscles requires standardized protocols of assessment and specific training of operators. Adequate reproducibility might be achieved once these criteria are met.

### Contrast-enhanced ultrasound (CEUS) and elastography

The microcirculation of the skeletal muscle is the primary and most important site for capillary-tissue exchange of nutrients, oxygen, and hormones, particularly during exercise.^104^ CEUS is used to assess the degree of muscle vascularization. Reduced blood flow to the muscles, caused by microvascular damage and decreased nitric oxide production, has been cited as a significant factor in the development of sarcopenia.^105,106^ Mitchell *et al*. have noted that the SonoVue contrast medium can be used to demonstrate a reduced circulatory response in muscle tissue to a nutritional stimulus, which may indirectly indicate a reduction in vascularization.^107^ Further research on patients with sarcopenia is needed to assess the utility of this method in a clinical setting, which may be diminished due to the need for using a contrast agent.^15,59^

Elastography is based on the change of the muscle’s biomechanical properties due to the increased content of fibrous and adipose tissue as well as glycated products.^108^ By measuring the change in muscle stiffness, information regarding muscle function (strength, power, range of motion) can be obtained.^39^ Shear wave sonoelastography, in contrast to strain mode, seems to have emerged as the dominant and superior modality for assessing muscle stiffness.^109,110^ Alfuraih *et al*. suggested that changes in muscle stiffness, detected by elastography, might be correlated to muscle weakness.^111^

Due to conflicting research results, it remains unclear whether muscle stiffness increases or decreases with age.^112–114^ Janczyk *et al*. conducted a systematic review to explore the potential of sonoelastography as a reliable method for assessing sarcopenia in older adults. Their findings indicate that the passive elastic constant was significantly higher in sarcopenic individuals compared to healthy subjects after passive stretching. Among the ten studies reviewed, four reported increased muscle stiffness in older adults, two reported decreased stiffness, and four found no significant differences. Ultimately, the authors could not draw definitive conclusions about the usefulness of elastography in assessing sarcopenia.^115^ Bastijns *et al*. propose that differences in passive torque, activity levels and the ultrasound probe axis may account for changes in shear values in different age groups.^110^ Standardisation of measurement protocols is paramount for effective comparison of studies using elastography in sarcopenia assessment.

### Ultrasound measurement protocol

As of now, there is no universally accepted standardized approach for conducting muscle ultrasonography in clinical practice. In 2018, The European Geriatric Medicine Society (EuGMS) SARCUS (SARCopenia through UltraSound) working group published their first recommendations on the standardization of the use of ultrasound for muscle assessment. In 2021, SARCUS proposed an updated consensus protocol for using ultrasound in muscle assessment, including measurement of muscle thickness, cross-sectional area, fascicle length, pennation angle and echogenicity.^39^

Both the EWSGOP2 and the SARCUS working groups emphasize that ultrasound has proven to be an accurate and reliable technique of muscle mass measurement in different populations, while showing high repeatability. However, standardization of measurement methods is paramount to perform extensive and comparative studies.^8,39^ The following paragraphs highlight the important aspects regarding standardisation of measurement protocols.

Due to its size, accessibility and comfort for the patient, US assessment of muscle mass is most often performed at the anterior compartment of the thigh. However, standardized anatomical landmarks and measuring points have now been proposed for 39 muscles/muscle groups.^39^ Standardization of ultrasound measurement points is vital due to the absence of definitive data regarding the extent of heterogeneity in ultrasound parameters across the muscle bulk.^116^

According to recommendations, muscles should be assessed in a relaxed state. Patients should refrain from any physical activity for at least thirty minutes and should remain in a lying position for the last five minutes prior to examination.^39,117^ This is important since physical activity or changing the body’s position may affect the fluid distribution and in turn the measured parameters.^117^

Studies have shown that the position of the patient as well as head of bed elevation during ultrasound assessment can significantly affect measurements, therefore using a standardized technique regarding positioning is vital during the examination.^118^ For follow up measurements, the same position should be used as during the first exam.

Depending on the muscle/muscle group being measured, patients should be examined in either supine or prone position. In clinical practice, some patients might find it difficult to lie in a prone position. Therefore, a sitting position, with knees and ankles bent in 90°, may be used to examine certain muscle groups, e.g. lower leg muscles and muscles of the head and neck.^39^

Muscle fiber pennation angle and fascicle length should be measured while keeping the transducer probe perpendicular to the longitudinal axis of the muscle. Cross-sectional area and echo intensity should be determined from images obtained by the probe being placed parallel to the longitudinal muscle axis. Muscle thickness can in practice be measured using either probe placement. Some parameters, such as muscle thickness, can easily be measured during the examination, while others (e.g. echo intensity) will need to be additionally analysed post examination. The US images can be analysed using various open-source scientific image processing programs, e.g. ImageJ (https://imagej.nih.gov/ij).

A high-frequency linear ultrasound probe (5–10 MHz) is usually recommended for muscle assessment. A minimum transducer length of 5 cm seems advisable to visualise as much tissue as possible, particularly in subjects with a larger muscle bulk. Standard B-mode should be applied to visualize the different muscle components. A copious amount of ultrasound gel should be used to avoid excessive pressure on the muscle, which could affect the measurements. It is advised to keep the probe as perpendicular to the skin surface as possible.

Ultrasound system settings can be set to have the best possible view of the muscle that is to be assessed. However, since these settings may significantly affect measurements of certain parameters (particularly echo intensity), settings should be standardised for study purposes as well as for follow up examinations in the clinical setting, particularly for specific muscles/muscle groups. This is particularly important for follow up examinations and in research settings, to produce reliable and comparable results.

There is no consensus on whether the muscles of the dominant or non-dominant side of the patients are to be assessed. Studies have reported measurements from both dominant and nondominant sides, while other studies did not report this information. Since it is not clear whether the measurement side has clinical relevance, it is paramount for future research purposes and analysis to clearly indicate which side was assessed.

### Ultrasound assessment of muscle function

The accuracy of ultrasound parameters in assessing muscle function has been a subject of numerous studies. Assessment of muscle function presents one of the cornerstones of sarcopenia diagnosis. While muscle strength can be used as a strong indicator of sarcopenia, the deterioration of physical performance may serve as an indicator of disease severity. Therefore, determining the reliability of ultrasound parameters in assessing muscle function may provide further opportunity to incorporate ultrasound in sarcopenia diagnosis.

A significant association between ultrasound-measured forearm muscle thickness and hand grip strength was demonstrated in both young and elderly volunteers.^119,120^ Other authors found a significant correlation between hand grip strength and ultrasound measurements of lower limb muscles as well.^121^ Seymour *et al*. demonstrated a positive correlation between rectus femoris cross-sectional area and knee extensor strength in COPD patients.^63^

Ismail *et al*. found that ultrasound measurements of muscle quality were more strongly correlated with muscle strength than DXA-determined muscle mass.^122^ Thomaes *et al*. found significant correlation between maximal quadriceps muscle strength and ultrasound-measured muscle thickness of rectus femoris in patients with coronary artery disease.^32^

In a study on 26 young and 26 older individuals, Strasser *et al*. reported a highly significant correlation between ultrasound-measured muscle thickness of quadriceps femoris muscles and maximal knee extensor strength. Muscle echogenicity was significantly higher in the older group. While no correlation was found between quadriceps muscle echogenicity and knee extensor strength in the older group, a negative correlation was found in the young cohort. Similarly, a correlation between pennation angle and knee extensor strength was found only in the vastus intermedius muscle in young individuals.^78^

Conversely, Fukumoto *et al*. found that ultrasound-measured quadriceps femoris echo-intensity in a group of 92 older healthy Japanese women was negatively correlated with muscle thickness and knee extensor isometric strength, while a positive correlation between muscle thickness and knee extensor strength was demonstrated.^123^ Similarly, Watanabe *et al*. demonstrated an inverse association between echo intensity of the quadriceps muscle and knee extension strength in elderly men.^124^ Wilheim *et al*. also reported significant negative correlations between echo intensity of the quadriceps femoris muscles and both knee extension strength as well as the 30 second sit-to stand test in 50 healthy men.^125^ Hirasawa *et al*. also found an association between echo intensity of the vastus lateralis muscle and knee extension strength in patients with type 2 diabetes.^126^

The clinical correlations between site-specific muscle loss and physical performance remains poorly understood. A study on older communitydwelling women conducted by Abe *et al*. showed that an age-related loss of adductor/quadriceps muscles may be associated with a decrease in performance of more difficult tasks, such as zig-zag walking, yet is not significantly correlated with gait speed.^127^ Similarly, Madden *et al*. found no correlation between vastus medialis muscle thickness and subject gait speed.^128^ On the other hand, Mateos-Angulo *et al*. showed a significant negative correlation between echo intensity of lower limb muscles and gait speed as well as short physical performance battery test in older adults.^129^ Osawa *et al*. concluded that echo intensity, but not muscle thickness, was associated with physical activity and functional mobility scores in the very elderly.^130^

In a scoping review, Kitagawa *et al*. found poor to moderate associations between muscle echo intensity measurements of various muscles and functional performance tests. The authors concluded that the accurate effect size and causal inferences between muscle echo intensity and functional performance remained unclear.^131^ A meta-analysis of twenty-eight studies by Yuan *et al*. demonstrated moderate to strong correlations between echo intensity, muscle thickness and cross-sectional area with muscle strength. The authors also reported no significant association between ultrasound parameters and gait speed or Timed Up and Go test. Weak correlation was reported between echo intensity and muscle thickness with the sit-to-stand test.^132^ In another meta-analysis, Oranchuk *et al*. demonstrated the strongest correlation between quadriceps femoris echo intensity and knee extensor strength.^133^

As of now, it is not yet clear whether any ultrasound parameter can be used as a strong indicator for physical performance. However, based on current research, qualitative parameters, such as echo intensity, might indeed be proven to be superior in this regard.

### Limitations and challenges of ultrasound assessment

Despite having numerous advantages compared to other diagnostic methods, the use of ultrasound in muscle assessment still presents with several limitations. A large proportion of research in the field of US muscle assessment has been done on healthy populations, which does not necessarily reflect the dynamics of muscle parameter changes in different patient populations. Additionally, ultrasound parameters regarding muscle quality and quantity do not have clearly defined reference values. To establish this, large-scale longitudinal studies both on healthy people and patient cohorts should be done, which would enable satisfactory differentiation between normal and sarcopenic states. Third, muscle quantity and quality values could be subject to change, particularly due to systemic inflammation, muscle damage or changes in fluid balance, vascular permeability or glycogen levels.^134,135^ Additionally, the exact spreading pattern and evolution of the different architectural components throughout the muscle is still unclear, as well as the exact influence of pre-investigation physical activity and patient position on the accuracy of measurements. Also, examiners require a certain level of experience and/or training. Intraand inter-rater reliability has been a subject of concern, particularly for muscle quality parameters (e.g. echo intensity), while large muscles, such as the quadriceps, are quite easily quantitatively assessed. The influence of ultrasound system settings on measurements should also be considered.

Furthermore, it is still unclear which muscle group parameters correlate best with overall muscle quantity and quality. While a lot of research has been focused on large muscle groups, smaller muscles have received relatively little attention yet may be of equal interest due to their specific functions.^39^ Additionally, while most of the research focused on the diagnostic utility of ultrasound parameters of superficial muscles, some studies have included measurements of deeper muscles, most commonly vastus intermedius and the soleus muscle. The depth of muscle tissue being assessed may influence the accuracy of measurements, particularly of muscle quality (e.g. echo intensity). Therefore, several authors have encouraged the use of a correction factor considering subcutaneous fat thickness, although results using this method have been conflicting. In obese patients with a deeper layer of subcutaneous fat, the ultrasound image depth and focus may need to be adapted to improve visualisation, and the use of correction factors should be considered. At the same time, it is paramount to establish standardisation of both ultrasound system settings and methodology of measurements.

Furthermore, the validity of ultrasound-derived prediction equations for the estimation of muscle mass in older adults is also yet to be established, due to the lack of definitive results from studies.^9^ Therefore, it is vital to harmonize ultrasound measurement protocols and establish cutoff values for sarcopenia diagnosis, which would also provide more reliable metadata in the future.

## Conclusions

Ultrasound muscle examination is a safe, accessible, and reliable method which has the potential of becoming a valuable tool in the diagnosis of sarcopenia. As the population continues to age, finding accurate and reliable methods for assessing muscle mass and muscle quality will become increasingly important. Early sarcopenia detection would enable more effective treatment and thus help reduce morbidity and mortality in these patients. Similarly, since sarcopenia is a proven negative prognostic factor for postoperative recovery, the implementation of a novel method for simple and non-invasive assessment of muscle tissue would enable a wider screening of patients at risk of sarcopenia and enable the adjustment of pre-operative preparations, treatment and rehabilitation, thereby improving treatment results.

To date, studies regarding agreement between ultrasound parameters and reference methods have shown that ultrasound is a potentially accurate diagnostic tool for sarcopenia detection, particularly when using muscle quantity parameters of lower extremities. Due to the heterogeneity of studies regarding ultrasound assessment of muscle quality, no definitive conclusions can yet be made, although it seems certain ultrasound parameters might prove accurate in assessing muscle function.

In general, the inclusion of ultrasound in the clinical practice of muscle assessment in patients with sarcopenia might potentially improve risk stratification and facilitate clinical decision-making regarding the need of nutritional and other interventions.

There are still several limitations that preclude the implementation of the ultrasound method in muscle assessment in everyday clinical practice. However, with growing interest in the utility of ultrasound in sarcopenia diagnosis, along with increasing availability of ultrasound, future studies, preferably on larger cohorts of both healthy volunteers and different patient groups, could clarify remaining uncertainties and provide definitive evidence regarding the utility of this promising method.

## References

[1] Dent E, Morley JE, Cruz-Jentoft AJ, Arai H, Kritchevsky SB, Guralnik J (2018). International Clinical Practice Guidelines for Sarcopenia (ICFSR): screening, diagnosis and management. J Nutr Health Aging.

[2] Shafiee G, Keshtkar A, Soltani A, Ahadi Z, Larijani B, Heshmat R. (2017). Prevalence of sarcopenia in the world: a systematic review and meta-analysis of general population studies. J Diabetes Metab Disord.

[3] Cederholm T, Barazzoni R, Austin P, Ballmer P, Biolo G, Bischoff SC (2017). ESPEN guidelines on definitions and terminology of clinical nutrition. Clin Nutr.

[4] Filippin LI, Teixeira VN de O, da Silva MPM, Miraglia F, da Silva FS. (2015). Sarcopenia: a predictor of mortality and the need for early diagnosis and intervention. Aging Clin Exp Res.

[5] Janssen I, Shepard DS, Katzmarzyk PT, Roubenoff R. (2004). The healthcare costs of sarcopenia in the United States. J Am Geriatr Soc.

[6] Moisey LL, Mourtzakis M, Cotton BA, Premji T, Heyland DK, Wade CE (2013). Skeletal muscle predicts ventilator-free days, ICU-free days, and mortality in elderly ICU patients. Crit Care.

[7] Rizzoli R, Reginster JY, Arnal JF, Bautmans I, Beaudart C, Bischoff-Ferrari H (2013). Quality of life in sarcopenia and frailty. Calcif Tissue Int.

[8] Cruz-Jentoft AJ, Bahat G, Bauer J, Boirie Y, Bruyère O, Cederholm T (2019). Sarcopenia: revised European consensus on definition and diagnosis. Age Ageing.

[9] Nijholt W, Scafoglieri A, Jager-Wittenaar H, Hobbelen JSM, van der Schans CP. (2017). The reliability and validity of ultrasound to quantify muscles in older adults: a systematic review. J Cachexia Sarcopenia Muscle.

[10] Beaudart C, Reginster JY, Slomian J, Buckinx F, Dardenne N, Quabron A (2015). Estimation of sarcopenia prevalence using various assessment tools. Exp Gerontol.

[11] Batsis JA, Barre LK, Mackenzie TA, Pratt SI, Lopez-Jimenez F, Bartels SJ. (2013). Variation in the prevalence of sarcopenia and sarcopenic obesity in older adults associated with different research definitions: dual-energy x-ray absorptiometry data from the National Health and Nutrition Examination Survey 1999–2004. J Am Geriatr Soc.

[12] Bijlsma AY, Meskers CG, Ling CH, Narici M, Kurrle SE, Cameron ID (2013). Defining sarcopenia: the impact of different diagnostic criteria on the prevalence of sarcopenia in a large middle aged cohort. Age (Dordr).

[13] Sanada K, Kearns CF, Midorikawa T, Abe T. (2006). Prediction and validation of total and regional skeletal muscle mass by ultrasound in Japanese adults. Eur J Appl Physiol.

[14] Mitsiopoulos N, Baumgartner RN, Heymsfield SB, Lyons W, Gallagher D, Ross R. (1998). Cadaver validation of skeletal muscle measurement by magnetic resonance imaging and computerized tomography. J Appl Physiol (1985).

[15] Ticinesi A, Meschi T, Narici MV, Lauretani F, Maggio M. (2017). Muscle ultrasound and sarcopenia in older individuals: a clinical perspective. J Am Med Dir Assoc.

[16] Pretorius A, Keating JL. (2008). Validity of real time ultrasound for measuring skeletal muscle size. Phys Ther Rev.

[17] Abe T, Thiebaud RS, Loenneke JP, Young KC. (2015). Prediction and validation of DXA-derived appendicular lean soft tissue mass by ultrasound in older adults. Age (Dordr).

[18] Hull H, He Q, Thornton J, Javed F, Allen L, Wang J (2009). iDXA, Prodigy, and DPXL dual-energy X-ray absorptiometry whole-body scans: a crosscalibration study. J Clin Densitom.

[19] Takai Y, Ohta M, Akagi R, Kato E, Wakahara T, Kawakami Y (2013). Validity of ultrasound muscle thickness measurements for predicting leg skeletal muscle mass in healthy Japanese middle-aged and older individuals. J Physiol Anthropol.

[20] Scafoglieri A, Deklerck R, Tresignie J, De Mey J, Clarys JP, Bautmans I. (2013). Assessment of regional adipose tissue depots: a DXA and CT comparison in cadavers of elderly persons. Exp Gerontol.

[21] Álvarez MN, Ronda MAV, Rangel LS, Thuissard-Vasallo IJ, Andreu-Vazquez C, Martin PM (2021). Muscle assessment by ultrasonography: agreement with dual-energy x-ray absorptiometry (DXA) and relationship with physical performance. J Nutr Health Aging.

[22] Dehghan M, Merchant AT. (2008). Is bioelectrical impedance accurate for use in large epidemiological studies?. Nutr J.

[23] Rösler A, Lehmann F, Krause T, Wirth R, von Renteln-Kruse W. (2010). Nutritional and hydration status in elderly subjects: clinical rating versus bioimpedance analysis. Arch Gerontol Geriatr.

[24] Goodpaster BH, Park SW, Harris TB, Kritchevsky SB, Nevitt M, Schwartz AV (2006). The loss of skeletal muscle strength, mass, and quality in older adults: the health, aging and body composition study. J Gerontol A Biol Sci Med Sci.

[25] Mitchell WK, Williams J, Atherton P, Larvin M, Lund J, Narici M. (2012). Sarcopenia, dynapenia, and the impact of advancing age on human skeletal muscle size and strength; a quantitative review. Front Physiol.

[26] Maddocks M, Jones M, Snell T, Connolly B, de Wolf-Linder S, Moxham J (2014). Ankle dorsiflexor muscle size, composition and force with ageing and chronic obstructive pulmonary disease. Exp Physiol.

[27] Barbat-Artigas S, Rolland Y, Zamboni M, Aubertin-Leheudre M. (2012). How to assess functional status: a new muscle quality index. J Nutr Health Aging.

[28] Newman AB, Haggerty CL, Goodpaster B, Harris T, Kritchevsky S, Nevitt M (2003). Strength and muscle quality in a well-functioning cohort of older adults: the health, aging and body composition study. J Am Geriatr Soc.

[29] Amini B, Boyle SP, Boutin RD, Lenchik L. (2019). Approaches to assessment of muscle mass and myosteatosis on computed tomography: a systematic review. J Gerontol A Biol Sci Med Sci.

[30] Aubrey J, Esfandiari N, Baracos VE, Buteau FA, Frenette J, Putman CT (2014). Measurement of skeletal muscle radiation attenuation and basis of its biological variation. Acta Physiol (Oxf).

[31] Heymsfield SB, Gonzalez MC, Lu J, Jia G, Zheng J. (2015). Skeletal muscle mass and quality: evolution of modern measurement concepts in the context of sarcopenia. Proc Nutr Soc.

[32] Thomaes T, Thomis M, Onkelinx S, Coudyzer W, Cornelissen V, Vanhees L. (2012). Reliability and validity of the ultrasound technique to measure the rectus femoris muscle diameter in older CAD-patients. BMC Med Imaging.

[33] Reeves ND, Maganaris CN, Narici MV. (2004). Ultrasonographic assessment of human skeletal muscle size. Eur J Appl Physiol.

[34] Cartwright MS, Demar S, Griffin LP, Balakrishnan N, Harris JM, Walker FO. (2013). Validity and reliability of nerve and muscle ultrasound. Muscle Nerve.

[35] Betz T, Wehrstein M, Preisner F, Bendszus M, Friedmann-Bette B. (2021). Reliability and validity of a standardised ultrasound examination protocol to quantify vastus lateralis muscle. J Rehabil Med.

[36] Janssen I, Heymsfield SB, Wang Z, Ross R. (2000). Skeletal muscle mass and distribution in 468 men and women aged 18–88 yr. J Appl Physiol (1985).

[37] Abe T, Sakamaki M, Yasuda T, Bemben MG, Kondo M, Kawakami Y (2011). Age-related, site-specific muscle loss in 1507 Japanese men and women aged 20 to 95 years. J Sports Sci Med.

[38] Abe T, Kawakami Y, Kondo M, Fukunaga T. (2011). Comparison of ultrasound-measured age-related, site-specific muscle loss between healthy Japanese and German men. Clin Physiol Funct Imaging.

[39] Perkisas S, Bastijns S, Baudry S, Bauer J, Beaudart C, Beckwée D (2021). Application of ultrasound for muscle assessment in sarcopenia: 2020 SARCUS update. Eur Geriatr Med.

[40] Mirón Mombiela R, Vucetic J, Rossi F, Tagliafico AS. (2020). Ultrasound biomarkers for sarcopenia: what can we tell so far?. Semin Musculoskelet Radiol.

[41] Fu H, Wang L, Zhang W, Lu J, Yang M. (2023). Diagnostic test accuracy of ultrasound for sarcopenia diagnosis: a systematic review and meta-analysis. J Cachexia Sarcopenia Muscle.

[42] Nijholt W, Jager-Wittenaar H, Raj IS, van der Schans CP, Hobbelen H. (2020). Reliability and validity of ultrasound to estimate muscles: a comparison between different transducers and parameters. Clin Nutr ESPEN.

[43] Hammond K, Mampilly J, Laghi FA, Goyal A, Collins EG, McBurney C (2014). Validity and reliability of rectus femoris ultrasound measurements: comparison of curved-array and linear-array transducers. J Rehabil Res Dev.

[44] Thoirs K, English C. (2009). Ultrasound measures of muscle thickness: intra-examiner reliability and influence of body position. Clin Physiol Funct Imaging.

[45] Cartwright MS, Demar S, Griffin LP, Balakrishnan N, Harris JM, Walker FO. (2013). Validity and reliability of nerve and muscle ultrasound. Muscle Nerve.

[46] Cagnie B, Derese E, Vandamme L, Verstraete K, Cambier D, Danneels L. (2009). Validity and reliability of ultrasonography for the longus colli in asymptomatic subjects. Man Ther.

[47] Dupont A, Sauerbrei EE, Fenton PV, Shragge PC, Loeb GE, Richmond FJR. (2001). Real-time sonography to estimate muscle thickness: comparison with MRI and CT. J Clin Ultrasound.

[48] Peres LM, Luis-Silva F, Menegueti MG, Lovato WJ, Espirito Santo DAD, Donadel MD (2024). Comparison between ultrasonography and computed tomography for measuring skeletal muscle mass in critically ill patients with different body mass index. Clin Nutr ESPEN.

[49] Abe T, Nakatani M, Loenneke JP. (2018). Relationship between ultrasound muscle thickness and MRI-measured muscle cross-sectional area in the forearm: a pilot study. Clin Physiol Funct Imaging.

[50] Miyachi R, Kanazawa Y, Fujii Y, Ohno N, Miyati T, Yamazaki T. (2022). Reliability of lower leg muscle thickness measurement along the long axis of the muscle using ultrasound imaging, in a sitting position. J Phys Ther Sci.

[51] Dhariwal S, Roy A, Taneja S, Bansal A, Gorsi U, Singh S (2023). Assessment of sarcopenia using muscle ultrasound in patients with cirrhosis and sarcopenic obesity (AMUSE STUDY). J Clin Gastroenterol.

[52] Zhao R, Li X, Jiang Y, Su N, Li J, Kang L (2022). Evaluation of appendicular muscle mass in sarcopenia in older adults using ultrasonography: a systematic review and meta-analysis. Gerontology.

[53] Staempfli JS, Kistler-Fischbacher M, Gewiess J, Bastian J, Eggimann AK. (2024). The validity of muscle ultrasound in the diagnostic workup of sarcopenia among older adults: a scoping review. Clin Interv Aging.

[54] Nies I, Ackermans LLGC, Poeze M, Blokhuis TJ, Ten Bosch JA. (2022). The diagnostic value of ultrasound of the rectus femoris for the diagnosis of sarcopenia in adults: a systematic review. Injury.

[55] Li L, Xia Z, Zeng X, Tang A, Wang L, Su Y. (2024). The agreement of different techniques for muscle measurement in diagnosing sarcopenia: a systematic review and meta-analysis. Quant Imaging Med Surg.

[56] Linek P. (2018). The importance of body mass normalisation for ultrasound measurements of the morphology of oblique abdominis muscles: the effect of age, gender, and sport practice. Folia Morphol (Warsz).

[57] Merrigan JJ, White JB, Hu YE, Stone JD, Oliver JM, Jones MT. (2018). Differences in elbow extensor muscle characteristics between resistance-trained men and women. Eur J Appl Physiol.

[58] Kanehisa H, Ito M, Kawakami Y, Fukunaga T, Miyatani M. (2004). The accuracy of volume estimates using ultrasound muscle thickness measurements in different muscle groups. Eur J Appl Physiol.

[59] Perkisas S, Baudry S, Bauer J, Beckwée D, De Cock AM, Hobbelen H (2018). Application of ultrasound for muscle assessment in sarcopenia: towards standardized measurements. Eur Geriatr Med.

[60] Aagaard P, Andersen JL, Dyhre-Poulsen P, Leffers AM, Wagner A, Magnusson SP (2001). A mechanism for increased contractile strength of human pennate muscle in response to strength training: changes in muscle architecture. J Physiol.

[61] Franchi MV, Longo S, Mallinson J, Quinlan JI, Taylor T, Greenhaff PL (2018). Muscle thickness correlates to muscle cross-sectional area in the assessment of strength training-induced hypertrophy. Scand J Med Sci Sports.

[62] Formenti P, Umbrello M, Coppola S, Froio S, Chiumello D. (2019). Clinical review: peripheral muscular ultrasound in the ICU. Ann Intensive Care.

[63] Seymour JM, Ward K, Sidhu PS, Puthucheary Z, Steier J, Jolley CJ (2009). Ultrasound measurement of rectus femoris cross-sectional area and the relationship with quadriceps strength in COPD. Thorax.

[64] Ozturk Y, Koca M, Burkuk S, Unsal P, Dikmeer A, Oytun MG (2022). The role of muscle ultrasound to predict sarcopenia. Nutrition.

[65] de Luis Roman D, García Almeida JM, Bellido Guerrero D, Guzmán Rolo G, Martín A, Primo Martín D (2024). Ultrasound cut-off values for rectus femoris for detecting sarcopenia in patients with nutritional risk. Nutrients.

[66] Looijaard WG, Dekker IM, Stapel SN, Girbes AR, Twisk JW, Oudemans-van Straaten HM (2016). Skeletal muscle quality as assessed by CT-derived skeletal muscle density is associated with 6-month mortality in mechanically ventilated critically ill patients. Crit Care.

[67] Body S, Ligthart MAP, Rahman S, Ward J, May-Miller P, Pucher PH (2022). Sarcopenia and myosteatosis predict adverse outcomes after emergency laparotomy. Ann Surg.

[68] Blackwell JEM, Herrod PJJ, Doleman B, Boyd-Carson H, Dolan D, Wheldon L (2023). CT-derived measures of muscle quantity and quality predict poorer outcomes from elective colorectal surgery: a UK multicentre retrospective cohort study. Tech Coloproctol.

[69] Kamiliou A, Lekakis V, Chrysavgis L, Cholongitas E. (2024). Prevalence and impact on the outcome of myosteatosis in patients with cirrhosis: a systematic review and meta-analysis. Hepatol Int.

[70] Lortie J, Rush B, Osterbauer K, Colgan TJ, Tamada D, Garlapati S (2022). Myosteatosis as a shared biomarker for sarcopenia and cachexia using MRI and ultrasound. Front Rehabil Sci.

[71] Reimers K, Reimers CD, Wagner S, Paetzke I, Pongratz DE. (1993). Skeletal muscle sonography: a correlative study of echogenicity and morphology. J Ultrasound Med.

[72] Wilhelm EN, Rech A, Minozzo F, Radaelli R, Botton CE, Pinto RS. (2014). Relationship between quadriceps femoris echo intensity, muscle power, and functional capacity of older men. Age (Dordr).

[73] Pillen S, van Keimpema M, Nievelstein RAJ, Verrips A, van Kruijsbergen-Raijmann W, Zwarts MJ. (2006). Skeletal muscle ultrasonography: visual versus quantitative evaluation. Ultrasound Med Biol.

[74] Watanabe Y, Ikenaga M, Yoshimura E, Yamada Y, Kimura M. (2018). Association between echo intensity and attenuation of skeletal muscle in young and older adults: a comparison between ultrasonography and computed tomography. Clin Interv Aging.

[75] Isaka M, Sugimoto K, Yasunobe Y, Akasaka H, Fujimoto T, Kurinami H (2019). The usefulness of an alternative diagnostic method for sarcopenia using thickness and echo intensity of lower leg muscles in older males. J Am Med Dir Assoc.

[76] Yamada M, Kimura Y, Ishiyama D, Nishio N, Abe Y, Kakehi T (2017). Differential characteristics of skeletal muscle in community-dwelling older adults. J Am Med Dir Assoc.

[77] Yoshida T, Kumon Y, Takamatsu N, Nozaki T, Inoue M, Nodera H (2022). Ultrasound assessment of sarcopenia in patients with rheumatoid arthritis. Mod Rheumatol.

[78] Strasser EM, Draskovits T, Praschak M, Quittan M, Graf A. (2013). Association between ultrasound measurements of muscle thickness, pennation angle, echogenicity and skeletal muscle strength in the elderly. Age (Dordr).

[79] Burton AM, Stock MS. (2018). Consistency of novel ultrasound equations for estimating percent intramuscular fat. Clin Physiol Funct Imaging.

[80] Valera-Calero JA, Arias-Buría JL, Fernández-de-las-Peñas C, Cleland JA, Gallego-Sendarrubias GM, Cimadevilla-Fernández-Pola E. (2021). Echo-intensity and fatty infiltration ultrasound imaging measurement of cervical multifidus and short rotators in healthy people: a reliability study. Musculoskelet Sci Pract.

[81] Mongold SJ, Georgiev C, Naeije G, Vander Ghinst M, Stock MS, Bourguignon M. (2024). Age-related changes in ultrasound-assessed muscle composition and postural stability. Sci Rep.

[82] Fukumoto Y, Taniguchi M, Hirono T, Yagi M, Yamagata M, Nakai R (2022). Influence of ultrasound focus depth on the association between echo intensity and intramuscular adipose tissue. Muscle Nerve.

[83] Girts RM, Harmon KK, Pagan JI, Alberto A, Hernandez MG, Stock MS. (2022). The influence of ultrasound image depth and gain on skeletal muscle echo intensity. Appl Physiol Nutr Metab.

[84] Paris MT, Bell KE, Avrutin E, Mourtzakis M. (2020). Ultrasound image resolution influences analysis of skeletal muscle composition. Clin Physiol Funct Imaging.

[85] Scafoglieri A, Van den Broeck J, Bartocci P, Cattrysse E, Jager-Wittenaar H, Gonzalez MC. (2024). Skeletal muscle echo intensity values differ significantly across ultrasound parameter settings. Life (Basel).

[86] Young H, Jenkins NT, Zhao Q, Mccully KK. (2015). Measurement of intramuscular fat by muscle echo intensity. Muscle Nerve.

[87] Blue MNM, Smith-Ryan AE, Trexler ET, Hirsch KR. (2018). The effects of high intensity interval training on muscle size and quality in overweight and obese adults. J Sci Med Sport.

[88] Stock MS, Oranchuk DJ, Burton AM, Phan DC. (2020). Age-, sex-, and regionspecific differences in skeletal muscle size and quality. Appl Physiol Nutr Metab.

[89] Neto Müller J, Lanferdini FJ, Passos Karam JY, de Brito Fontana H. (2021). Examination of the confounding effect of subcutaneous fat on muscle echo intensity utilizing exogenous fat. Appl Physiol Nutr Metab.

[90] Palmer TB, Farrow AC. (2022). Correcting for subcutaneous fat: does it improve the correlation between vastus lateralis echo intensity and physical performance in older women?. Clin Physiol Funct Imaging.

[91] Paris MT, Mourtzakis M. (2021). Muscle composition analysis of ultrasound images: a narrative review of texture analysis. Ultrasound Med Biol.

[92] Narici MV, Maganaris CN, Reeves ND, Capodaglio P. (2003). Effect of aging on human muscle architecture. J Appl Physiol (1985).

[93] Selva Raj I, Bird SR, Shield AJ. (2017). Ultrasound measurements of skeletal muscle architecture are associated with strength and functional capacity in older adults. Ultrasound Med Biol.

[94] Randhawa A, Wakeling JM. (2013). Associations between muscle structure and contractile performance in seniors. Clin Biomech (Bristol).

[95] Narici M, Franchi M, Maganaris C. (2016). Muscle structural assembly and functional consequences. J Exp Biol.

[96] Narici MV, Binzoni T, Hiltbrand E, Fasel J, Terrier F, Cerretelli P. (1996). In vivo human gastrocnemius architecture with changing joint angle at rest and during graded isometric contraction. J Physiol.

[97] Ando R, Nosaka K, Inami T, Tomita A, Watanabe K, Blazevich AJ (2016). Difference in fascicle behaviors between superficial and deep quadriceps muscles during isometric contractions. Muscle Nerve.

[98] Ema R, Wakahara T, Mogi Y, Miyamoto N, Komatsu T, Kanehisa H (2013). In vivo measurement of human rectus femoris architecture by ultrasonography: validity and applicability. Clin Physiol Funct Imaging.

[99] Infantolino BW, Challis JH. (2014). Short communication: pennation angle variability in human muscle. J Appl Biomech.

[100] Kruse NT, Hughes WE, Casey DP. (2018). Mechanistic insights into the modulatory role of the mechanoreflex on central hemodynamics using passive leg movement in humans. J Appl Physiol (1985).

[101] Blazevich AJ, Gill ND, Zhou S. (2006). Intra-and intermuscular variation in human quadriceps femoris architecture assessed in vivo. J Anat.

[102] Kuyumcu ME, Halil M, Kara Ö, Çuni B, Çağlayan G, Güven S (2016). Ultrasonographic evaluation of the calf muscle mass and architecture in elderly patients with and without sarcopenia. Arch Gerontol Geriatr.

[103] Narici M, McPhee J, Conte M, Franchi MV, Mitchell K, Tagliaferri S (2021). Age-related alterations in muscle architecture are a signature of sarcopenia: the ultrasound sarcopenia index. J Cachexia Sarcopenia Muscle.

[104] Dunford EC, Au JS, Devries MC, Phillips SM, MacDonald MJ. (2018). Cardiovascular aging and the microcirculation of skeletal muscle: using contrast-enhanced ultrasound. Am J Physiol Heart Circ Physiol.

[105] Morley JE, Anker SD, von Haehling S. (2014). Prevalence, incidence, and clinical impact of sarcopenia: facts, numbers, and epidemiology – update 2014. J Cachexia Sarcopenia Muscle.

[106] Marzetti E, Calvani R, Cesari M, Buford TW, Lorenzi M, Behnke BJ (2013). Mitochondrial dysfunction and sarcopenia of aging: from signaling pathways to clinical trials. Int J Biochem Cell Biol.

[107] Mitchell WK, Phillips BE, Williams JP, Rankin D, Smith K, Lund JN (2013). Development of a new SonovueTM contrast-enhanced ultrasound approach reveals temporal and age-related features of muscle microvascular responses to feeding. Physiol Rep.

[108] Drenth H, Zuidema S, Bunt S, Bautmans I, van der Schans C, Hobbelen H. (2016). The contribution of advanced glycation end product (AGE) accumulation to the decline in motor function. Eur Rev Aging Phys Act.

[109] Wang JC, Wu WT, Chang KV, Chen LR, Chi SY, Kara M (2021). Ultrasound imaging for the diagnosis and evaluation of sarcopenia: an umbrella review. Life (Basel).

[110] Bastijns S, De Cock AM, Vandewoude M, Perkisas S. (2020). Usability and pitfalls of shear-wave elastography for evaluation of muscle quality and its potential in assessing sarcopenia: a review. Ultrasound Med Biol.

[111] Alfuraih AM, Tan AL, O’Connor P, Emery P, Wakefield RJ. (2019). The effect of ageing on shear wave elastography muscle stiffness in adults. Aging Clin Exp Res.

[112] Akagi R, Yamashita Y, Ueyasu Y. (2015). Age-related differences in muscle shear moduli in the lower extremity. Ultrasound Med Biol.

[113] Yoshida K, Itoigawa Y, Maruyama Y, Saita Y, Takazawa Y, Ikeda H (2017). Application of shear wave elastography for the gastrocnemius medial head to tennis leg. Clin Anat.

[114] Eby SF, Cloud BA, Brandenburg JE, Giambini H, Song P, Chen S (2015). Shear wave elastography of passive skeletal muscle stiffness: influences of sex and age throughout adulthood. Clin Biomech (Bristol).

[115] Janczyk EM, Champigny N, Michel E, Raffaelli C, Annweiler C, Zory R (2021). Sonoelastography to assess muscular stiffness among older adults and its use for the diagnosis of sarcopenia: a systematic review. Ultraschall Med.

[116] Reeves ND, Narici MV, Maganaris CN. (2004). In vivo human muscle structure and function: adaptations to resistance training in old age. Exp Physiol.

[117] Lopez P, Pinto MD, Pinto RS. (2019). Does rest time before ultrasonography imaging affect quadriceps femoris muscle thickness, cross-sectional area and echo intensity measurements?. Ultrasound Med Biol.

[118] Hacker ED, Peters T, Garkova M. (2016). Ultrasound assessment of the rectus femoris cross-sectional area. West J Nurs Res.

[119] Abe T, Thiebaud RS, Loenneke JP, Ogawa M, Mitsukawa N. (2014). Association between forearm muscle thickness and age-related loss of skeletal muscle mass, handgrip and knee extension strength and walking performance in old men and women: a pilot study. Ultrasound Med Biol.

[120] Abe T, Counts BR, Barnett BE, Dankel SJ, Lee K, Loenneke JP. (2015). Associations between handgrip strength and ultrasound-measured muscle thickness of the hand and forearm in young men and women. Ultrasound Med Biol.

[121] Rech A, Radaelli R, Goltz FR, da Rosa LHT, Schneider CD, Pinto RS. (2014). Echo intensity is negatively associated with functional capacity in older women. Age (Dordr).

[122] Ismail C, Zabal J, Hernandez HJ, Woletz P, Manning H, Teixeira C (2015). Diagnostic ultrasound estimates of muscle mass and muscle quality discriminate between women with and without sarcopenia. Front Physiol.

[123] Fukumoto Y, Ikezoe T, Yamada Y, Tsukagoshi R, Nakamura M, Mori N (2012). Skeletal muscle quality assessed from echo intensity is associated with muscle strength of middle-aged and elderly persons. Eur J Appl Physiol.

[124] Watanabe Y, Yamada Y, Fukumoto Y, Ishihara T, Yokoyama K, Yoshida T (2013). Echo intensity obtained from ultrasonography images reflecting muscle strength in elderly men. Clin Interv Aging.

[125] Wilhelm EN, Rech A, Minozzo F, Radaelli R, Botton CE, Pinto RS. (2014). Relationship between quadriceps femoris echo intensity, muscle power, and functional capacity of older men. Age (Dordr).

[126] Hirasawa Y, Matsuki R, Tanina H. (2022). Relationship between echo intensity of vastus lateralis and knee extension strength in patients with type 2 diabetes mellitus. Phys Ther Res.

[127] Abe T, Ogawa M, Loenneke JP, Thiebaud RS, Loftin M, Mitsukawa N. (2012). Relationship between site-specific loss of thigh muscle and gait performance in women: the HIREGASAKI study. Arch Gerontol Geriatr.

[128] Madden KM, Feldman B, Arishenkoff S, Meneilly GS. (2021). A rapid point-of-care ultrasound marker for muscle mass and muscle strength in older adults. Age Ageing.

[129] Mateos-Angulo A, Galán-Mercant A, Cuesta-Vargas AI. (2021). Muscle thickness and echo intensity by ultrasonography and cognitive and physical dimensions in older adults. Diagnostics (Basel).

[130] Osawa Y, Arai Y, Oguma Y, Hirata T, Abe Y, Azuma K (2017). Relationships of muscle echo intensity with walking ability and physical activity in the very old population. J Aging Phys Act.

[131] Kitagawa T, Nakamura M, Fukumoto Y. (2023). Usefulness of muscle echo intensity for evaluating functional performance in the older population: a scoping review. Exp Gerontol.

[132] Yuan H, Kim MK. (2024). Exploring the relationship between ultrasound parameters and muscle strength in older adults: a meta-analysis of sarcopeniarelated exercise performance. Front Med (Lausanne).

[133] Oranchuk DJ, Bodkin SG, Boncella KL, Harris-Love MO. (2024). Exploring the associations between skeletal muscle echogenicity and physical function in aging adults: a systematic review with meta-analyses. J Sport Health Sci.

[134] Stock MS, Thompson BJ. (2021). Echo intensity as an indicator of skeletal muscle quality: applications, methodology, and future directions. Eur J Appl Physiol.

[135] Welch C, Greig CA, Hassan-Smith ZK, Pinkney TD, Lord JM, Jackson TA. (2019). A pilot observational study measuring acute sarcopenia in older colorectal surgery patients. BMC Res Notes.

